# Cystatin C as Biomarker for the Evaluation of Renal Outcome in AL Amyloidosis

**DOI:** 10.1002/ajh.27716

**Published:** 2025-05-19

**Authors:** Foteini Theodorakakou, Despina Fotiou, Filia Apostolakou, Ioannis Papassotiriou, Vasiliki Spiliopoulou, Ioannis Ntanasis‐Stathopoulos, Panagiotis Malandrakis, Magdalini Migkou, Nikolaos Kanellias, Evangelos Eleutherakis‐Papaiakovou, Erasmia Psimenou, Asimina Papanikolaou, Charikleia Gakiopoulou, Smaragdi Marinaki, Stavroula Giannouli, Maria Gavriatopoulou, Evangelos Terpos, Meletios‐Athanasios Dimopoulos, Efstathios Kastritis

**Affiliations:** ^1^ Department of Clinical Therapeutics National and Kapodistrian University of Athens Athens Greece; ^2^ Department of Clinical Biochemistry “Aghia Sophia” Children's Hospital Athens Greece; ^3^ First Department of Pediatrics, School of Medicine National and Kapodistrian University of Athens Athens Greece; ^4^ Department of Haemopathology Evangelismos Hospital Athens Greece; ^5^ First Department of Pathology National and Kapodistrian University of Athens, School of Medicine Athens Greece; ^6^ Clinic of Nephrology and Renal Transplantation National and Kapodistrian University of Athens, School of Medicine, Laikon Hospital Athens Greece; ^7^ Second Department of Internal Medicine, School of Medicine National and Kapodistrian University of Athens Athens Greece; ^8^ Department of Medicine Korea University Seoul South Korea

**Keywords:** cystatin C, dialysis, glomerular filtration rate, kidney, light chain amyloidosis

## Abstract

Cystatin C (CysC) has emerged as a novel and potentially more reliable biomarker for the estimation of glomerular filtration in the general population in patients with various conditions. In AL amyloidosis, the current renal staging system and renal response criteria are based on proteinuria and creatinine‐based eGFR. We explored the prognostic role of CysC and of estimation of eGFR based on CysC‐based equations in a cohort of 195 patients with newly diagnosed AL amyloidosis with renal involvement. Baseline CysC level was strongly and independently associated with progression to dialysis, and CysC levels ≥ 1.9 mg/L can be used in combination with the current renal staging system to identify patients with different risk of progression to dialysis among renal stages 2 and 3. eGFR based on CysC performed at least similarly to eGFR based on creatinine alone (by CKD‐EPI race free formula) and the cutoff of 30 mL/min/1.73 m^2^ could better predict progression to dialysis at 2 years. At 6 months landmark, an increase in CysC by ≥ 1 mg/L was associated with higher risk of progression to dialysis (HR: 19.8, 95% CI 6.5–60.5, *p* < 0.001); a reduction of CysC based eGFR ≥ 30% was also associated with poor renal outcome, with a prognostic performance similar to current renal progression criteria. In conclusion, CysC provides prognostic information regarding the renal outcomes in patients with AL amyloidosis independently of the established biomarkers, but requires further validation.

## 1 Introduction

Renal amyloidosis is encountered in almost 70% of patients with immunoglobulin light chain (AL) amyloidosis. Advanced renal disease can have a detrimental effect on patients' quality of life, with a high risk of progression to end‐stage renal disease requiring dialysis. The staging system that is currently used to stratify patients into three stages is based on creatinine‐based estimated glomerular filtration rate (eGFR) and 24‐h proteinuria [1]. Considering that eGFR and proteinuria can vary significantly, affected by age, diet, muscle mass, concomitant medications, patient adherence to proper 24‐h urine collection, and considering that renal response, defined as a reduction in proteinuria by at least 30% from baseline, is delayed compared with hematologic response, there is an unmet need for more accurate tools to assess renal outcome.

Cystatin C (CysC) is an emerging endogenous marker of kidney function and is considered to be a more reliable biomarker to estimate glomerular filtration than creatinine. New equations to estimate GFR have been published lately that use either CysC alone (with or without the inclusion of race) or a combination of creatinine and CysC [2, 3], and it is now recommended to use the eGFR equation that requires both creatinine and CysC because it represents the most precise estimation of GFR.

In the current study, we evaluated the potential prognostic impact of CysC and as a surrogate biomarker for renal outcome in patients with newly diagnosed AL amyloidosis.

## 2 Methods

### 2.1 Patients

The analysis included 195 consecutive unselected patients with newly diagnosed AL amyloidosis with renal involvement, treated in the department of Clinical Therapeutics in Athens, Greece. All patients had a histopathological diagnosis of AL amyloidosis, and organ involvement was defined according to ISA consensus criteria [4, 5]. Renal stages were calculated as stage 1 if eGFR was ≥ 50 mg/mL/1.73 m^2^ and proteinuria was ≤ 5000 mg/24 h, stage 2 if either eGFR was > 50 mg/mL/1.73 m^2^ or proteinuria was > 5000 mg/24 h, and stage 3 if both eGFR was > 50 ml/mL/1.73 m^2^ and proteinuria > 5000 mg/24 h, according to the established stratification system [1]. For the assessment of renal and hematologic response, standard criteria were used [1, 6]. All patients had available serum samples at diagnosis and before they received treatment, and 109 patients had also serum samples at 6‐month landmark from the initiation of treatment. All patients gave written consent for blood sampling and for analyzing their medical data. After venipuncture, serum was separated within 4 h and stored at −80°C for all patients. Clinical data were prospectively registered in a locally maintained database. Patients were treated according to our institution's practice. Serum free light chains were measured using the FreeLite assay (The Binding Site, San Diego).

### 2.2 Measurement of Cystatin C

CysC was measured with the assay by Roche Diagnostics CYSC2 225T, a fully automated particle‐enhanced immunoturbidimetric assay for CysC in undiluted serum and EDTA‐plasma. Human CysC agglutinates with latex particles coated with anti‐CysC antibodies. The aggregate is determined turbidimetrically at 552 nm. The assay is reported to have normal ranges between 0.61 and 0.95 mg/L. Roche Diagnostics did not provide any financial support and did not participate in the design of the study, serum measurements, analysis, or interpretation of the results, or manuscript preparation. Measurements were performed at the Department of Clinical Biochemistry, “Aghia Sophia” Children's Hospital.

### 2.3 eGFR Calculations

We calculated eGFR based on creatinine using the Modification of Diet in Renal Disease (MDRD) formula [7] and the latest equations from the Chronic Kidney Disease Epidemiology Collaboration (CKD‐EPI) [2]. We calculated eGFR based on CysC or a combination of both creatinine and CysC, using the latest equations from the CKD‐EPI. We also calculated eGFR based on CysC, without the inclusion of sex based on the European Kidney Function Consortium (EKFC) formula [2, 3, 8]. The equations are shown in Table S1. Although we tested both eGFR equations based on creatinine (MDRD and CKD‐EPI), here we report the results based on CKD‐EPI, which is the most widely used formula today. In Data S1, data about eGFR based on the MDRD equation are also presented.

### 2.4 Statistical Analysis

Time to dialysis or renal survival (RS) was calculated from the time of initial diagnosis until the time of dialysis initiation or the time of last follow‐up or the time of death, whatever occurred first. Overall survival (OS) was calculated from the time of initial diagnosis until the time of death or the time of last follow‐up, if it occurred first. Time to event curves were plotted with Kaplan–Meier and comparisons were made using the log‐rank test with *p* values < 0.05 considered statistically significant. Univariate and multivariate analyses were performed using the Cox regression. Receiver operating characteristic (ROC) analyses based on progression to dialysis at 2 years identified the thresholds of baseline variables best predicting renal survival. SPSS software was used (IBM SPSS Statistics, version 29, IBM Corp., Armonk, NY).

## 3 Results

Baseline disease and treatment characteristics are shown in Table S2. Median baseline cystatin C (CysC) levels were 1.53 mg/L (range 0.58–7.18 mg/L; quartiles 0.58–1.11 mg/L, 1.11–1.53 mg/L, 1.53–2.2 mg/L, 2.2–7.18 mg/L) and 87.7% of the patients had CysC levels more than the upper limit for the assay (normal range 0.61–0.95 mg/L). Palladini Renal stage distribution for stage 1, 2, and 3 was 27%, 53%, and 20%; among patients with baseline CysC levels < 0.95 mg/L (UNL) renal stage distribution was 37%, 63%, and 0% for stages 1, 2, and 3, while among patients with baseline CysC levels ≥ 0.95 mg/L 25%, 52%, and 23% of patients were renal stages 1, 2, and 3 respectively.

### 3.1 Correlation of Cystatin C With Baseline Characteristics

There was a significant and strong correlation of baseline CysC with baseline serum creatinine (Spearman *r* = 0.826, *p* < 0.001) and CKD‐EPI eGFRcr (Spearman *r* = −0.834, *p* < 0.001) (Figure S1A,B). Baseline median CysC levels in patients with renal stages 1, 2, and 3 were 1.35 mg/L (range 0.63–7.18 mg/L), 1.4 mg/L (range 0.67–3.97), and 2.85 (1.49–5.64 mg/L), respectively, being similar for stages 1 and 2 (*p* = 0.612) but significantly higher for stage 3 (*p* < 0.001) (Figure S2). There was no correlation between serum CysC levels and the level of proteinuria (*p* = 0.431) or with serum albumin levels (*p* = 0.071). A correlation of CysC with NT‐proBNP (Spearman *r* = 0.468, *p* < 0.001), hsTnT (Spearman *r* = 0.432, *p* < 0.001) and alkaline phosphatase (*r* = 0.202, *p* = 0.005) was observed. Consequently, CysC level was associated with heart involvement (*p* < 0.001) and Mayo stage (*p* < 0.001). It has been shown that CysC may also be secreted by plasma cells [9], but we found no correlation with clone characteristics such as plasma cell burden in the bone marrow (*p* = 0.091), free light chain levels (*p* = 0.533), presence of translocation t(11;14) (*p* = 0.972) or amplification 1q (*p* = 0.190).

### 3.2 Hematologic and Renal Outcomes

The median follow‐up time for the whole cohort was 55.4 months. Among evaluable patients (*n* = 180), at 1‐month landmark (*n* = 179), the overall hematologic response rate was 73.5% (≥ VGPR 53% and PR 20.5%); at 3‐month landmark (*n* = 172), the overall hematologic response was 80% (≥ VGPR 68% and PR 12%), and at 6‐month landmark (*n* = 159), the overall hematologic response was 86% (≥ VGPR 78% and PR 8%). On ITT, the overall hematologic response rate at primary therapy was 78% (≥ VGPR 64% and PR 14%); 81 patients (41.5%) received salvage therapy. There was no significant association of baseline CysC levels with depth or probability of hematologic response.

According to the Palladini criteria [1], renal response rate at 3‐ and 6‐month landmarks was 19% and 25.6%, and renal PD rate was 21% and 15.9%.

During follow up, 43 patients (22%) progressed to end stage renal disease requiring dialysis. The 1‐, 2‐, 3‐, and 5‐year dialysis rate was 13%, 16%, 18%, and 25% (Table S3). Per Palladini renal stage, the 2‐year dialysis rate was 3%, 15%, and 55%; 8 of the 43 (19%) patients progressed to dialysis more than 5 years after initial diagnosis.

### 3.3 Prognostic Significance of Baseline Cystatin for Renal Survival

**FIGURE 1:**
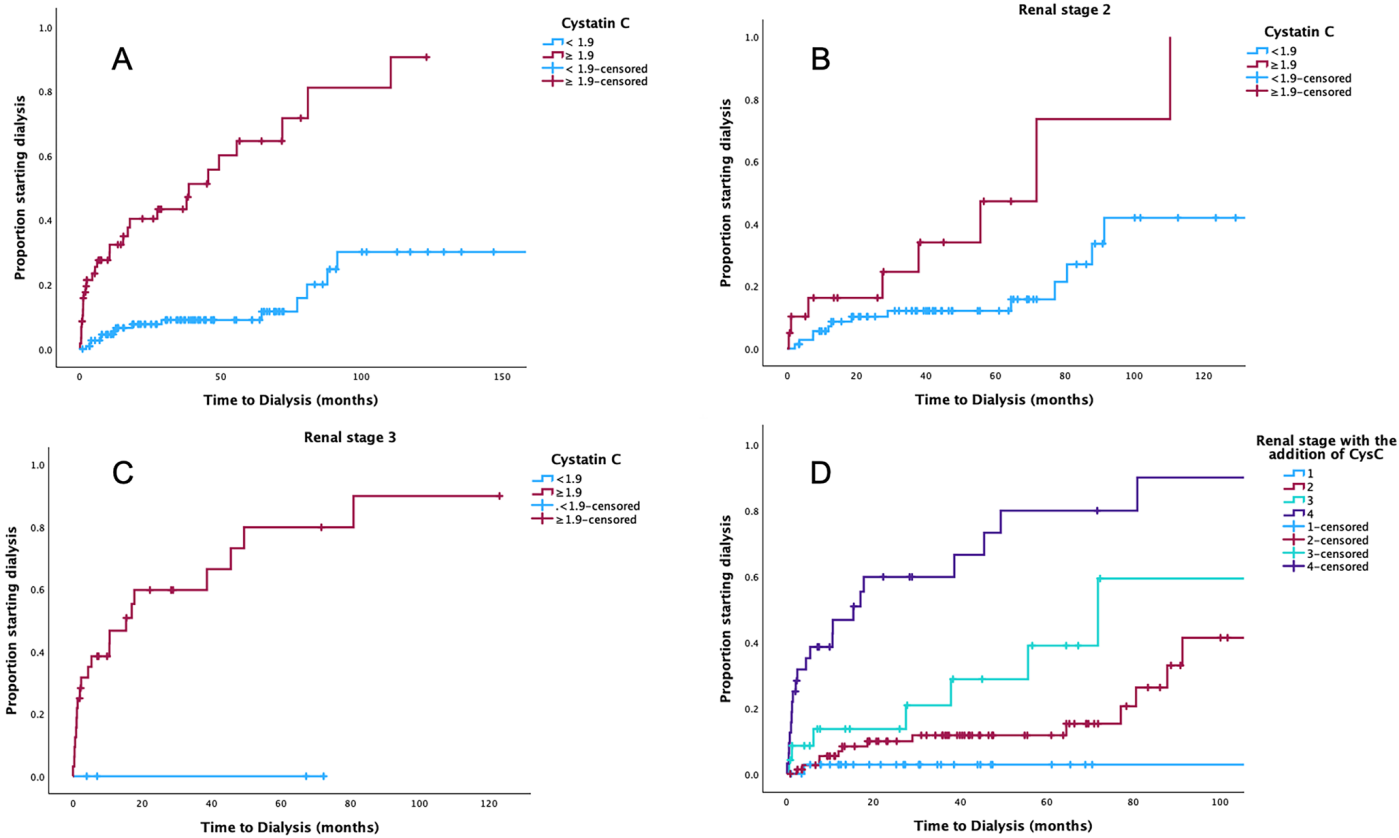
(Using eGFR CKD‐EPI for renal stage). Time to dialysis according to baseline Cystatin C levels using the cutoff of 1.9 mg/L for all patients (A), for patients with renal stage 2 (B) and renal stage 3 (C) and according to a new renal system with the addition of Cystatin C ≥ 1.9 mg/L. [Color figure can be viewed at wileyonlinelibrary.com]

Higher CysC level was associated with shorter time to dialysis, as a continuous variable (HR 2.537, 95% CI 2.010–3.203, *p* < 0.001) (Table 1). Using ROC analysis with the outcome need for dialysis at 2 years, a CysC level > 1.9 mg/L was associated with higher risk (sensitivity 0.714 and specificity 0.743; HR 7.159, 95% CI 3.747–13 679, *p* < 0.001); renal survival has not been reached for patients with CysC < 1.9 mg/L and was 38.6 months for patients with CysC ≥ 1.9 mg/L (*p* < 0.001) (Figure 1A). Because this level of cystatin is significantly higher than the ULN and to avoid overfitting, we also evaluated the prognostic impact of CysC > 0.95 mg/L (the upper limit of normal for the assay) which was not associated with a significant increase in the risk of progression to dialysis (HR 2.960, 95% CI 0.910–9.628, *p* = 0.071). Other factors that predicted time to dialysis, in univariate analysis, are also shown in Table 1. In the Table S3, dialysis rate according to CysC levels is depicted.

**TABLE 1:** Univariate analysis for time to dialysis and multivariate analysis including renal stage and Cystatin C as continuous variable (Model 1) and as categorical variable (Models 2 and 3).

Variable			
Univariate analysis	HR	95% CI	*p*
Age, years	1.022	0.991–1.054	0.173
Female	0.458	0.241–0.872	**0.018**
Light chain type	1.030	0.504–1.109	0.935
dFLC, mg/L	1	0.999–1	0.405
dFLC ≥ 180 mg/L	0.921	0.487–1.740	0.800
BMPC, %	1.004	0.987–1.021	0.657
BMPC ≥ 20%	1.501	0.928–2.403	0.090
Creatinine, mg/dL	1.837	1.593–2.118	**< 0.001**
eGFR CKD‐EPI, mL/min/1.73 m^2^	0.969	0.958–0.979	**< 0.001**
eGFR CKD‐EPI < 50 mL/min/1.73 m^2^	5.014	2.670–9.418	**< 0.001**
Proteinuria > 5000 mg/24 h	2.688	1.192–6.063	**0.017**
Serum albumin, g/dL	0.861	0.577–1.286	0.466
Renal stage 1	1	1	
Renal stage 2	9.361	1.257–69.691	**0.029**
Renal stage 3	42.437	5.683–316.841	**< 0.001**
Cystatin C baseline, mg/L	2.537	2.010–3.203	**< 0.001**
Cystatin C > 1.9 mg/L	7.159	3.747–13.679	**< 0.001**
eGFRcys, mL/min/1.73 m^2^	0.954	0.937–0.971	**< 0.001**
eGFRcys < 50 mL/min/1.73 m^2^	3.879	1.791–8.399	**< 0.001**
eGFRcys^a^ < 30 mL/min/1.73 m^2^	5.829	3.141–10.819	**< 0.001**
eGFRcr‐cys mL/min/1.73 m^2^	0.960	0.946–0.974	**< 0.001**
eGFRcr‐cys < 50 mL/min/1.73 m^2^	4.957	2.479–9.914	**< 0.001**
eGFRcr‐cys^a^ < 30 mL/min/1.73 m^2^	6.909	3.747–12.708	**< 0.001**
eGFRcys‐ns, mL/min/1.73 m^2^	0.949	0.931–0.967	**< 0.001**
eGFRcys‐ns < 50 mL/min/1.73 m^2^	4.361	2.067–9.201	**< 0.001**
eGFRcys‐ns^a^ < 30 mL/min/1.73 m^2^	5.726	3.108–10.551	**< 0.001**

Multivariate analysis			
Model 1			
Renal stage 1	1 (ref)		
Renal stage 2	7.222	0.965–54.062	0.054
Renal stage 3	14.177	1.771–113.509	**0.012**
Cystatin C	2.011	1.509–2.682	**< 0.0001**
Model 2			
Renal stage 1	1 (ref)		
Renal stage 2	7.864	1.052–58.784	**0.045**
Renal stage 3	15.209	1.891–122.299	**0.010**
Cystatin C > 1.9 mg/L	3.881	1.755–8.582	**< 0.0001**
Model 3			
Proteinuria > 5000 mg/24 h	2.588	1.141–5.866	**0.023**
eGFR CKD‐ EPI < 50 mL/min/1.73 m^2^	1.942	0.686–5.498	0.211
Cystatin C > 1.9 mg/L	3.997	1.384–11.540	**0.010**

Abbreviations: BMPC, bone marrow plasma cell; dFLC, difference between involved and uninvolved free light chains; eGFR, estimated glomerular filtration rate. *p* < 0.05 values are considered statistically significant.

^a^ Cutoffs defined by ROC analysis for dialysis at 2 years.

We then performed a multivariable analysis which included renal stage and CysC, which showed that cystatin level was an independent prognostic factor of progression to dialysis. The results were similar when using the 1.9 mg/L cutoff for CysC (Table 1). We also evaluated a model that included the individual components of the Palladini renal staging system, in which only the level of proteinuria > 5 g/day and cystatin > 1.9 mg/L remained significant but not eGFR < 50 mL/min/1.73 m^2^ (Table 1).

CysC level could further discriminate risk for ESRD among patients with renal stage 2 (2‐, 3‐, and 5‐year dialysis rate 7%, 11%, and 12% vs. 17%, 27%, and 25%, *p* = 0.040) and among patients with renal stage 3 (those with cystatin < 1.9 mg/L (*N* = 4) had a very low probability of progression to dialysis at 2‐years, 0% vs. 61%, *p* = 0.05) (Figure 1B,C).

Thus, by adding CysC ≥ 1.9 mg/L in the Palladini renal stage as an additional risk factor, we could have a 4‐stage stratification able to identify a group with very high risk of progression to ESRD for which current treatment approaches are inadequate (Figure 1D).

### 3.4 Use of eGFR Calculated With Cystatin C or Combined Creatinine and Cystatin C

Equations to calculate eGFR based on CysC have been developed and validated in large cohorts [2, 3, 8]. Since CysC showed prognostic impact when added to the current renal staging system, we further evaluated the prognostic performance of creatinine‐based and CysC‐based calculated eGFR (and on both creatinine and CysC), as previously defined [2, 3, 7, 8].

There was a significant correlation of CKD‐EPI eGFR based on creatinine with eGFR based on CysC (*r* = 0.844, *p* < 0.001) and with eGFR based on both creatinine and CysC (*r* = 0.971, *p* < 0.001) but as shown in (Figure S1C) there was significant dispersion, with CysC‐based calculated eGFR underestimating eGFR compared with creatinine‐based eGFR. Thus, an eGFR < 50 mL/min/1.73 m^2^ was found in 33% of patients according to the eGFR based on creatinine, in 60% of patients according to eGFR based on CysC, in 54% according to eGFR based on both creatinine and CysC, and in 56% according to eGFR based on CysC without sex. The agreement in stage classification between eGFRcr and the CysC‐based equations was 71%, 85%, and 74%, respectively (Table S4). In univariate analysis, an eGFR < 50 mL/min/1.73 m^2^ calculated with any of the equations was associated with progression to dialysis (Table 1; Figure S3A–D).

The cutoff of 50 mL/min/1.73 m^2^ has been evaluated in different cohorts while evaluating optimal cutoffs for eGFR calculated by CysC in our cohort would be associated with data overfitting. Thus, we evaluated the optimal cutoff for eGFR based on creatinine by the CKD‐EPI formula in our cohort, acknowledging data overfitting for all the eGFR equations with and without cystatin. In our cohort, an eGFRcr cutoff of 26 mL/min/1.73 m^2^ was identified. Notably, a similar cutoff was identified for eGFRcys (30 mL/min/1.73 m^2^), for eGFRcr‐cys (30 mL/min/1.73 m^2^) and for eGFRcys‐ns (34 mL/min/1.73 m^2^). Thus, by using our cohort‐specific cutoffs (i.e., 30 mL/min/1.73 m^2^), all equations performed similarly, without significant differences in their ability to identify patients developing ESRD within 2 years (Table S5).

### 3.5 Prognostic Significance of Changes of Cystatin Levels

We subsequently evaluated the potential of CysC as a biomarker for renal response and progression compared with or if added to the standard criteria by Palladini et al. At 6 months, 17 patients had already progressed to dialysis and 14 had died due to cardiac amyloidosis. In 109 patients, there was an available serum sample at 6 months to measure CysC, and among them, 102 patients were evaluable for renal response. The median CysC level at 6 months, among evaluable patients for renal response, was 1.61 mg/L (range 0.8–4.24 mg/L). Among these evaluable patients, the renal progression rate was 28% and the renal response and stable renal function rates were 42% and 30%, respectively. Among patients with renal response, renal stable disease and renal progression at 6 months, the median CysC levels were 1.42 mg/L (0.8–3.75 mg/L), 1.35 mg/L (0.84–3.05 mg/L), and 2.09 mg/L (1.17–4.24 mg/L), respectively (*p* < 0.001). Compared with baseline, 31% of the patients had a reduced cystatin level (median −8%; range −0.5% to −34%) and 69% had increased levels (median 23%; range 1%–328%). Achieving a hematologic VGPR or better (recorded in 84% of evaluable patients) was associated with renal response (*p* = 0.024) and CysC levels at 6 months were lower in patients with hemVGPR or better (*p* = 0.040).

CysC level at 6 months was associated with progression to dialysis (HR 3.614, 95% CI 1.954–6.6682, *p* < 0.001); similarly, eGFRcr and CysC‐calculated eGFR were all associated with progression to dialysis as well as 6‐month serum albumin levels but not proteinuria levels (Table 2). A hematologic VGPR or better at 6 months (≥ VGPR) was also associated with improved renal survival (2‐year dialysis rate was 4% vs. 28% for patients with < VGPR) (Figure S4). Other factors that were associated with renal survival in univariate analysis are shown in Table 2. At the 6‐month landmark, renal PD by Palladini criteria was associated with shorter renal survival (*p* = 0.003); there was no difference in renal survival for patients with renal response or stable renal status.

**TABLE 2:** Univariate and multivariate analysis for time to dialysis at 6‐month landmark.

Variable			
Univariate analysis	HR	95% CI	*p*
Creatinine at 6 months, mg/dL	3.002	1.753–5.209	**< 0.001**
eGFR CKD‐EPI at 6 months, mL/min/1.73 m^2^	0.953	0.925–0.982	**0.002**
Proteinuria at 6 months, mg/24 h	1		0.322
Serum albumin at 6 months, g/dL	0.332	0.138–0.802	**0.014**
Renal PD at 6 months	5.549	1.555–19.803	**0.008**
Renal response at 6 months	0.123	0.016–0.967	**0.046**
Cystatin C at 6 months, mg/L	3.614	1.954–6.682	**< 0.001**
Cystatin C increase > 1 mg/L	19.809	6.521–60.495	**< 0.001**
eGFRcys at 6 months, mL/min/1.73 m^2^	0.928	0.887–0.972	**0.001**
eGFRcr‐cys at 6 months, mL/min/1.73 m^2^	0.949	0.915–0.984	**0.005**
eGFRcys‐ns at 6 months, mL/min/1.73 m^2^	0.926	0.885–0.969	**< 0.001**
Hem response at 6 months, CR/VGPR	0.222	0.103–0.482	**< 0.001**

Multivariate analysis			
Model 1			
Serum albumin at 6 months, g/dL	0.334	0.138–0.810	**0.015**
eGFR CKD‐EPI at 6 months, mL/min/1.73 m^2^	0.985	0.938–1.034	0.536
Cystatin C at 6 months, mg/L	2.637	0.817–8.514	**0.105**
Model 2			
Renal PD at 6 months	1.741	0.372–8.160	0.482
Cystatin C at 6 months, mg/L	3.091	1.477–6.466	**0.003**
Model 3			
Renal response at 6 months	1.091	0.297–4.006	0.895
Cystatin C at 6 months, mg/L	3.605	1.904–6.826	**< 0.001**
Model 4			
Renal PD at 6 months	2.227	0.462–10.748	0.319
Cystatin C baseline	2.585	1.140–5.864	**0.023**
Cystatin C increase > 1 mg/L	6.708	1.071–42.007	**0.042**

*Note: p* < 0.05 values are considered statistically significant.

In multivariate analysis, CysC level remained an independent predictor for renal survival when renal response or renal PD criteria were used in the model (Table 2).

**FIGURE 2:**
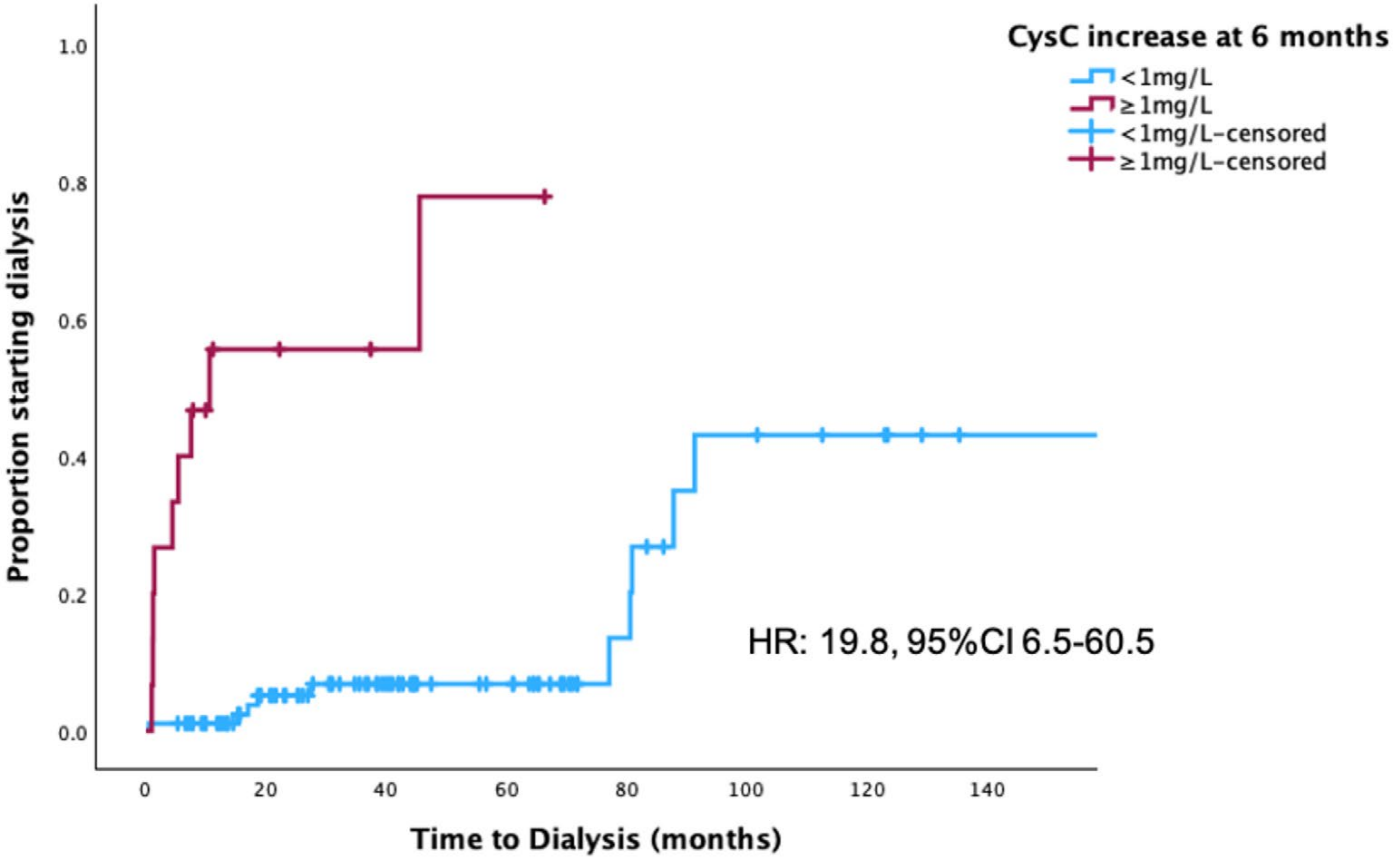
Time to dialysis at 6‐month landmark analysis in patients who had a Cystatin C increase ≥ 1 mg/L versus those without an increase or with a reduction. [Color figure can be viewed at wileyonlinelibrary.com]

Increases in the levels of CysC levels at 6 months (vs. baseline) were strongly associated with renal survival (*p* = 0.003). An optimal cut‐off increase of > 37% was associated with worse renal outcome; we used the 40% cutoff (observed in 26/109 (24%) patients) to evaluate the risk of progression to dialysis. Both renal PD by Palladini criteria and 6‐month CysC increase by 40% were associated with renal survival (Figure S5A,B), but 12% were progressors with both criteria, 34% with either, while 59% with neither. An absolute increase of 1 mg/L was strongly associated with progression to dialysis, even after adjusting for baseline CysC levels and for renal PD (HR: 19.8, 95% CI 6.5–60.5, *p* < 0.001) (Figure 2).

We evaluated whether eGFR calculated based on cystatin levels was more accurate to predict progression to dialysis at 2 years; although the AUC of eGFRcr‐cys was slightly higher, it did not differ significantly from that of eGFR calculated by creatinine alone or other CysC‐based equations (Table S6).

Since renal PD is based on eGFRcr reduction by at least 25%, we performed a ROC analysis to identify what changes in eGFR based on cystatin (calculated separately for each equation) could predict progression to dialysis at 2 years. For eGFRcr‐cys, the best cutoff was a 28% reduction. For eGFRcys, the best cutoff was a 34% reduction, and for eGFR based on CysC without sex, it was a 30% reduction from baseline. Thus, similar levels of reduction in eGFR, calculated by any equation, with or without cystatin, were associated with a higher 2‐year probability of progression to dialysis (Figure S6A–C).

### 3.6 Survival Analysis

Median OS for the whole cohort is estimated to be 92.6 months. In univariate analysis, higher baseline CysC levels were associated with inferior OS (HR 1.697, 95% CI 1.063–2.708, *p* = 0.027). Patients with CysC ≤ 1.9 mg/L had median OS of 112 months versus 80 months for patients with CysC > 1.9 mg/L (*p* = 0.025) (Figure S7). In multivariate analysis with Mayo stage, serum CysC did not have independent prognostic value.

## 4 Results Based on MDRD eGFR Formula

We have tested both eGFR equations based on creatinine (MDRD and CKD‐EPI) in comparison to CysC and eGFR equations based on CysC, and we have noticed similar results. In Data S1, data about eGFR based on the MDRD equation are also presented (Figure S8; Tables S7 and S8).

## 5 Discussion

In patients with AL amyloidosis, renal involvement is associated with a substantial increase in the risk of progression to end stage renal disease requiring dialysis, significant morbidity, and limitations in therapeutic options. Similar to cardiac biomarkers, proteinuria and eGFR are the two biomarkers that are used to risk stratify patients with renal AL involvement and to assess renal response and progression. The currently available criteria have certain limitations, and new biomarkers could be helpful. To this end, CysC may be a strong and independent prognostic factor for renal survival. To our knowledge, this is the first time that CysC has been used as a biomarker to predict renal outcome in patients with newly diagnosed AL amyloidosis. Two reports have been previously published in patients with AA amyloidosis and hereditary ATTR amyloidosis about the role of CysC in the evaluation of renal disease compared with creatinine and eGFR based on creatinine [10, 11], but the number of patients was small.

The established staging system and the criteria for renal response assessment have been validated in various cohort [12, 13, 14]. However, there are limitations, especially in the use of eGFR based on creatinine. Creatinine is not a reliable filtration marker in glomerular diseases [15]. What's more, in AL amyloidosis, patients often experience weight loss and have lower muscle mass, so creatinine and creatinine‐based eGFR calculations may not accurately reflect renal function [16, 17]. Therefore, alternative methods to evaluate renal function could be useful. We have previously proposed the use of the 24 h proteinuria to eGFR ratio as an alternative to the Palladini staging system and renal response criteria [12] and have been externally validated and compared, with similar performance to the established Palladini criteria; however, both are based on serum creatinine and 24 h proteinuria, having similar limitations [14].

Compared with creatinine, CysC rises earlier in acute kidney injury, has a shorter half‐life, is only distributed extracellularly, and is relatively freely filtered by the glomeruli, reabsorbed, and catabolized in the proximal tubule; thus, it may be a more accurate marker for the detection of early kidney damage [18]. Also, CysC is less affected by parameters such as muscle mass, race, gender, and age, which leads to higher sensitivity for kidney injury and a more precise estimation of glomerular filtration than creatinine [17, 19, 20, 21]. There are data that support that patients with lower cystatin C‐based eGFR than creatinine‐based eGFR, that is, patients with higher serum CysC, have worse prognosis [22]. In our cohort of patients with renal AL amyloidosis, CysC was elevated in most patients, even among patients with stage 1 renal disease, proving that it is a more sensitive biomarker of renal damage. CysC levels were associated with renal survival but, furthermore, they were an independent predictor of ESRD risk even after adjustment for renal stage or individual components of renal stage. Thus, CysC could identify a subgroup of patients with extremely dismal renal prognosis. This finding may have implications for the assessment of therapies that target renal amyloid and for the interpretation of renal outcomes from other trials.

Serum CysC levels at 6 months and changes from baseline were prognostic of renal outcomes; similarly, cystatin‐calculated eGFR changes were prognostic for renal survival, but in our cohort, they had a similar prognostic impact to eGFR based only on creatinine. This lack of superiority of CysC‐based eGFR evaluation may be due to the relatively small number of patients in our cohort, despite the fact that CysC levels alone were prognostic. Nonetheless, an increase in the level of CysC by 1 mg/L at 6 months was a very strong predictor of ESRD risk, independently of renal response status by standard criteria or baseline renal stage or eGFR level. We propose that such a strong predictor of renal outcome should be further evaluated, ideally in prospective trials.

Recently there is a shift toward the use of eGFR based on CysC or on both creatinine and CysC for the evaluation of chronic kidney disease in the general population and in various clinical conditions, although not widely adopted yet [23]. CysC has been, also, independently associated with cardiovascular disease and all‐cause mortality in the general population and in various conditions, including diabetes, coronary artery disease, and cirrhosis [19, 24, 25, 26, 27, 28]. CysC may be associated with inflammation [20] but this has been questioned [29, 30]. In contrast to creatinine, CysC levels rose in the hyperthyroid state [31], thus thyroid function should be considered when CysC is used. Another limitation for CysC is that it is one of the most highly up‐regulated genes in myeloma and it has been implicated as a marker for tumor burden; thus, it raises some concern about accuracy in plasma cell dyscrasias [9]. However, in our study we found no correlation of CysC with BMPC infiltration or FLCs.

Based on our findings, CysC is a simpler and possibly more accurate predictor of renal outcomes compared with eGFR based on CysC or creatinine (alone or in combination) at diagnosis and during the course of the disease. Thus, we recommend the use of CysC in combination with the established biomarkers to improve the stratification of patients, to identify patients at high risk of ending up in dialysis and to predict renal survival during the treatment course. Similarly, when observing response to therapy, the absolute increase of serum CysC compared with percentage changes seems to be more predictive of renal progression and could help guide treatment, especially for patients who have not achieved optimal hematologic response. The major limitation of our study is the relatively small number of patients, especially to assess renal response, and second, the lack of external validation. However, there is a strong signal that CysC may provide significant prognostic information beyond and over renal stage with clinical implications. Our purpose here is not to propose a new renal staging system or new response criteria but to investigate, for the first time, the prognostic role of CysC. The evaluation of CysC as a biomarker of renal response requires further validation and larger cohorts with adequate follow‐up, ideally in contemporary treated patients.

In conclusion, CysC is a sensitive biomarker of renal dysfunction in patients with renal AL amyloidosis with prognostic implications and further refines the current renal staging system. Further validation would define the potential role of CysC as a renal‐specific biomarker with usefulness in the prediction of outcome, risk stratification, and assessment of treatment response, especially in a changing landscape of anti‐clonal and anti‐amyloid therapies.

## Author Contributions

E.K. and F.T. designed the study, collected the data, performed the analysis and wrote the manuscript. I.P. and F.A. measured Cystatin C and revised the manuscript. E.K., F.T., D.F., V.S., I.N.‐S., P.M., M.M., N.K., E.E.‐P., E.P., A.P., C.G., S.M., S.G., M.G., E.T., and M.‐A.D. revised the manuscript.

## Ethics Statement

All subjects in the study were enrolled in IRB‐approved protocols at the respective institutions and the study was conducted in accordance with the Declaration of Helsinki.

## Consent

All patients provided oral and informed consent to use their data for the purpose of the study.

## Conflicts of Interest

D.F. declares honoraria from Sanofi and Janssen; M.M. declares honoraria from Janssen and GSK; I.N.‐S. declares honoraria from Janssen; M.G. declares consultancy and honoraria from Amgen, Abbvie, Integris, Karyopharm, Genesis Pharma, Janssen, GSK, Swixx and Takeda; E.T. declares consultancy and honoraria from BMS, Janssen, Celgene, Takeda, Genesis Pharma, Amgen, AstraZeneca, EUSA Pharma, GSK, Menarini/Stemline, Pfizer, BMS, Sanofi, Swixx, Antengen and Novartis; M.‐A.D. declares consultancy, boards and honoraria from Janssen, Celgene, Takeda, Amgen, BMS, Menarini, GSK, BeiGene, Swixx, AstraZeneca, Regeneron and Sanofi; E.K. declares consultancy, boards and honoraria from Abbvie, Genesis Pharma, Takeda, Janssen, Amgen, Pfizer, GSK, Sanofi and Prothena; F.T., I.P., F.A., V.S., P.M., N.K., E.E.‐P., E.P., A.P., C.G., S.M., and S.G. declare no financial interests.

## Supporting information


**Data S1.** Supporting Information.

## Data Availability

The data are available from the corresponding author upon reasonable request.
