# Supplementary material for: Cystatin C as Biomarker for the Evaluation of Renal Outcome in AL Amyloidosis
Source: Am J Hematol. 2025 May 19;100(8):1305–13. doi: 10.1002/ajh.27716 (PMC12232513; doi:10.1002/ajh.27716)
Supplement: Supplementary file 1 — Data S1. Supporting Information. [file AJH-100-1305-s001.docx]

**Cystatin as biomarker for the evaluation of renal outcome in AL amyloidosis – Supplementary material**

**Table S1.** Creatine-based and Cystatin C-based eGFR equations.

| MDRD^1^ eGFR |  |  | 186 x Scr^-1.154^ x age^-0.203^ [x 0.742 if female] |
| --- | --- | --- | --- |
| 2021 CKD-EPI eGFR^2^ | Female  Male | SCr ≤ 0.70  SCr > 0.70  SCr ≤ 0.90  SCr > 0.90 | 142 x (SCr/0.70)^-0.241^ x 0.9938^age^ x 1.012  142 x (SCr/0.70)^-1.200^ x 0.9938^age^ x 1.012  142 x (SCr/0.90)^-0.302^ x 0.9938^age^  142 x (SCr/0.90)^-1.200^ x 0.9938^age^ |
| 2012 CKD-EPI eGFRcys^3^ |  | ScysC ≤ 0.80  ScysC > 0.80 | 133 x (SCysC/0.80)^-0.499^ x 0.9962^age^ [x 0.932 if female]  133 x (SCysC/0.80)^-1.328^ x 0.9962^age^ [x 0.932 if female] |
| 2021 CKD-EPI eGFRcr-cys^2^ | Female  Male | SCr ≤ 0.70 ScysC ≤ 0.80  SCr ≤ 0.70 ScysC > 0.80  SCr > 0.70 ScysC ≤ 0.80  SCr > 0.70 ScysC > 0.80  SCr ≤ 0.90 ScysC ≤ 0.80  SCr ≤ 0.90 ScysC > 0.80  SCr > 0.90 ScysC ≤ 0.80  SCr > 0.90 ScysC > 0.80 | 135 x (SCr/0.70)^-0.219^ x (ScysC/0.80)^-0.323^ x 0.9961^age^ x 0.963  135 x (SCr/0.70)^-0.219^ x (ScysC/0.80)^-0.778^ x 0.9961^age^ x 0.963  135 x (SCr/0.70)^-0.544^ x (ScysC/0.80)^-0.323^ x 0.9961^age^ x 0.963  135 x (SCr/0.70)^-0.544^ x (ScysC/0.80)^-0.778^ x 0.9961^age^ x 0.963  135 x (SCr/0.90)^-0.144^ x (ScysC/0.80)^-0.323^ x 0.9961^age^  135 x (SCr/0.90)^-0.144^ x (ScysC/0.80)^-0.778^ x 0.9961^age^  135 x (SCr/0.90)^-0.544^ x (ScysC/0.80)^-0.323^ x 0.9961^age^  135 x (SCr/0.90)^-0.544^ x (ScysC/0.80)^-0.778^ x 0.9961^age^ |
| EKFC eGFRcys-ns^4^ | Age ≤40  Age >40 | ScysC/Q < 1.0  ScysC/Q ≥ 1.0  ScysC/Q < 1.0  ScysC/Q ≥ 1.0 | 107.3 x (SCysC/Q)^-0.322^  107.3 x (SCysC/Q)^-1.132^  107.3 x (SCysC/Q)^-0.322^ x 0.990^(age-40)^  107.3 x (SCysC/Q)^-1.132^ x 0.990^(age-40)^ |

Scr: serum creatinine (mg/dl)

SCysC: serum cystatin C (mg/L)

Q (for EKFC cystatin C-based eGFR): 0.83 [if age ≤50] and 0.83 + 0.005x(age−50) [if age >50].

**Table S2.** Baseline characteristics

|  | N=195 |
| --- | --- |
| Median age in years (range) | 65 (39-88) |
| Male / Female (%) | 53 / 47 |
| Organ involvement (%)  Heart  Renal  Liver  Nerve  Soft tissue | 69  100  17  23  21 |
| Number of organ involvement (range) | 2 (1-4) |
| Purpura (%) | 13 |
| Carpal tunnel syndrome (%) | 8 |
| Median BMPC, % (range) | 15 (0-80) |
| Median iFLC, mg/L (range) | 174 (9-9000) |
| Median dFLC, mg/L (range) | 142.3 (0.16-8987.8) |
| dFLC > 180 (%) | 44 |
| **Type of free light chain (%)**  kappa  lambda | 22  78 |
| **Mayo stage (%)**  1 / 2 / 3a / 3b | 22 / 44 / 24 / 10 |
| **Renal stage (%)**  1 / 2 / 3 | 27 / 53 / 20 |
| Median creatinine mg/dl (range) | 1.09 (0.40-8.9) |
| eGFR MDRD | 99 (5-178.15) |
| Median eGFR CKD-EPI, ml/min/1.73m^2^ (range) | 66.20 (4.98-119.01) |
| Median cystatin C, mg/L (range) | 1.53 (0.58-7.18) |
| Median eGFRcys ml/min/1.73m^2^ (range) | 41.78 (6.72-139.65) |
| Median eGFRcreat-cys ml/min/1.73m^2^ (range) | 54 (6-117) |
| Median eGFRcys-ns ml/min/1.73m^2^ (range) |  |
| Median proteinuria, g/24h (range) | 6451 (500-40000) |
| Proteinuria > 5000mg/24h (%) | 59 |
| eGFR<50 ml/min/1.73m^2^ (%) | 33 |
| Median serum albumin, g/L (range) | 3.0 (1.0-5.1) |
| Median NTproBNP, ng/L (range) | 2154 (33-75000) |
| Median hsTnT (range) | 38 (3-692) |
| Median SBP mmHg (range) | 112 (66-170) |
| Orthostasis (%) | 30 |
| Renal biopsy (%) | 48 |
| **Primary treatment (%)**  Bortezomib-containing regimens  Daratumumab-containing regimens  Lenalidomide-containing regimens  Melphalan  ASCT | 69  23.5  9  16  5 |
| **Cytogenetics (N=106 patients) (%)**  Translocation t(11;14)  Translocation t(14;16)  Translocation t(4;14)  Amplification or gain 1q21  Deletion 1p32  Deletion 13q  Deletion 17p | 47  4  4  29.5  2  34  4 |

**
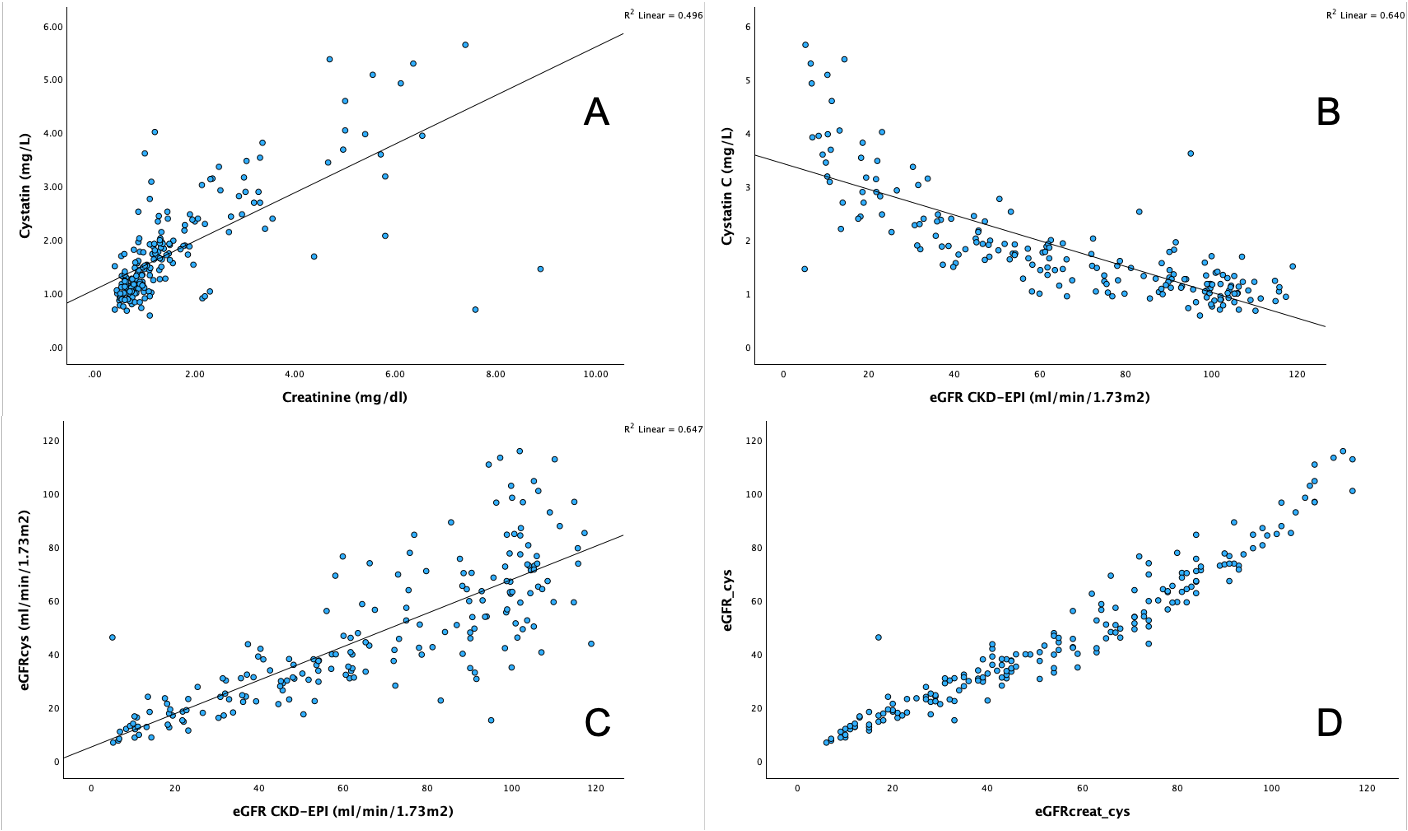
**

**Figure S1.** Correlation of baseline Cystatin C with creatinine (A) and eGFRcr CKD-EPI (B). Correlation of eGFRcys (C) and eGFRcr-cys (D) with eGFRcr CKD-EPI

**
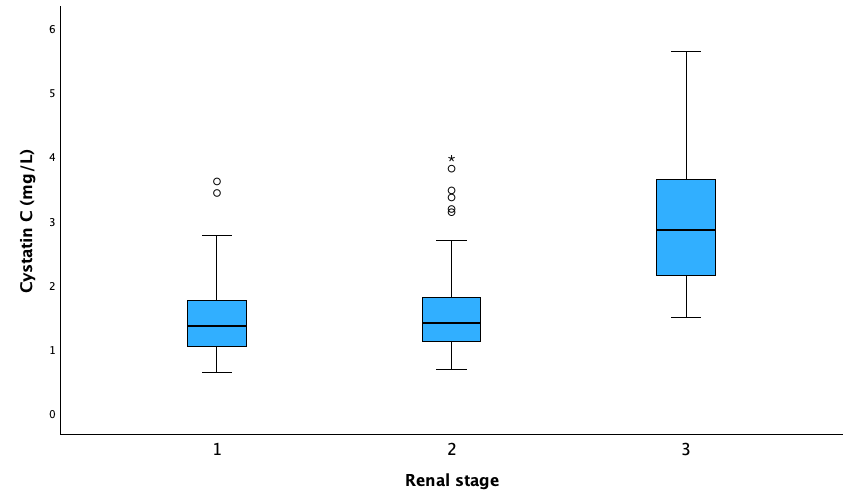
**

**Figure S2.** Baseline Cystatin C levels per Renal stage.

**
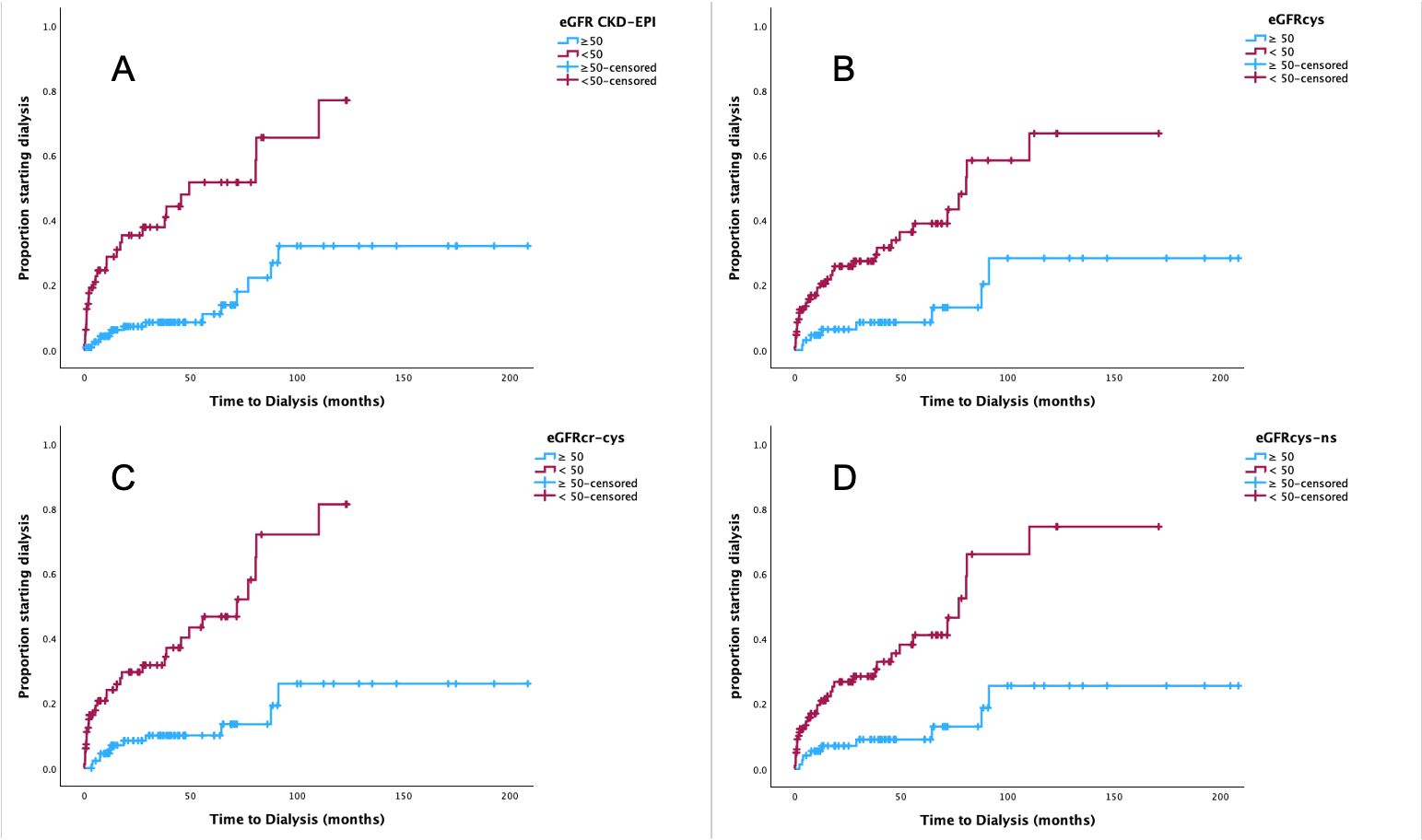
**

**Figure S3.** Time to dialysis in patients with eGFRcr <50 ml/min/1.73m^2^ calculated with CKD-EPI(A), eGFRcys (B), eGFRcr-cys (C) and eGFRcys-ns (D).

**Table S3.** Dialysis rate for all patients and according to Cystatin C levels.

|  | **1-year** | **2-year** | **3-year** | **5-year** |
| --- | --- | --- | --- | --- |
| All patients | 13% | 16% | 18% | 25% |
| Cystatin C < 0.95 mg/L | 0% | 0% | 7% | 7% |
| Cystatin C 0.95 – 1.9 mg/L | 7% | 10% | 10% | 10% |
| Cystatin C ≥ 1.9 mg/L | 33% | 40% | 44% | 65% |

**Table S4.** Reclassification of patients to renal stages using proteinuria >5000 mg/24h and all four equations for eGFR<50 ml/min/1.73m^2^.

|  | **Renal stage 1** | **Renal stage 2** | **Renal stage 3** |
| --- | --- | --- | --- |
| Proteinuria & eGFRcr CKD-EPI | 26% | 53% | 21% |
| Proteinuria & eGFRcys | 14% | 50% | 36% |
| Proteinuria & eGFRcr-cys | 17% | 55% | 28% |
| Proteinuria & eGFRcys-ns | 15% | 51% | 34% |

**Table S5.** ROC analysis for prognostic performance of eGFR< 30 mL/min/1.73m^2^ with all four equations of eGFR.

|  | **ROC AUC** | **Std. Err.** | **95% CI** |
| --- | --- | --- | --- |
| eGFRcr CKD-EPI <30 mL/min/1.73m^2^ | 0.754 | 0.058 | 0.641-0.867 |
| eGFRcys <30 mL/min/1.73m^2^ | 0.703 | 0.056 | 0.593-0.814 |
| eGFRcr-cys <30 mL/min/1.73m^2^ | 0.741 | 0.056 | 0.630-0.851 |
| eGFRcys-ns <30 mL/min/1.73m^2^ | 0.689 | 0.059 | 0.574-0.805 |


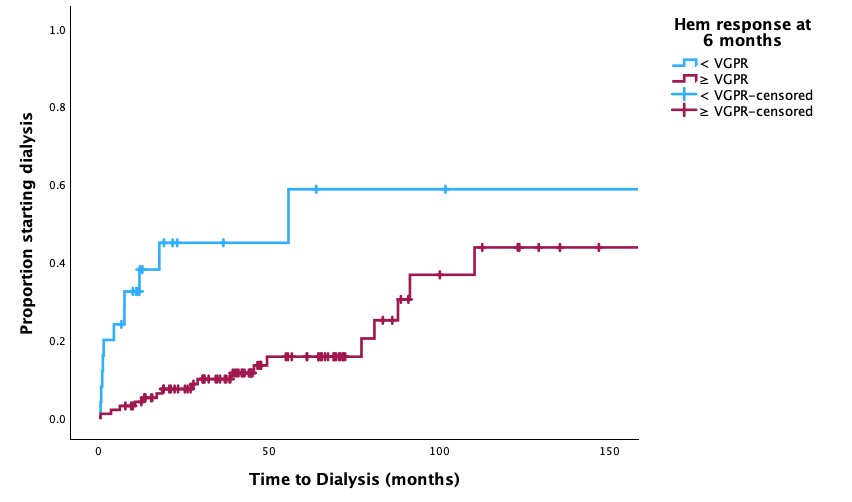


**Figure S4.** 6-momh landmark analysis in patients who had achieved at least VGPR vs those who had less than VGPR.


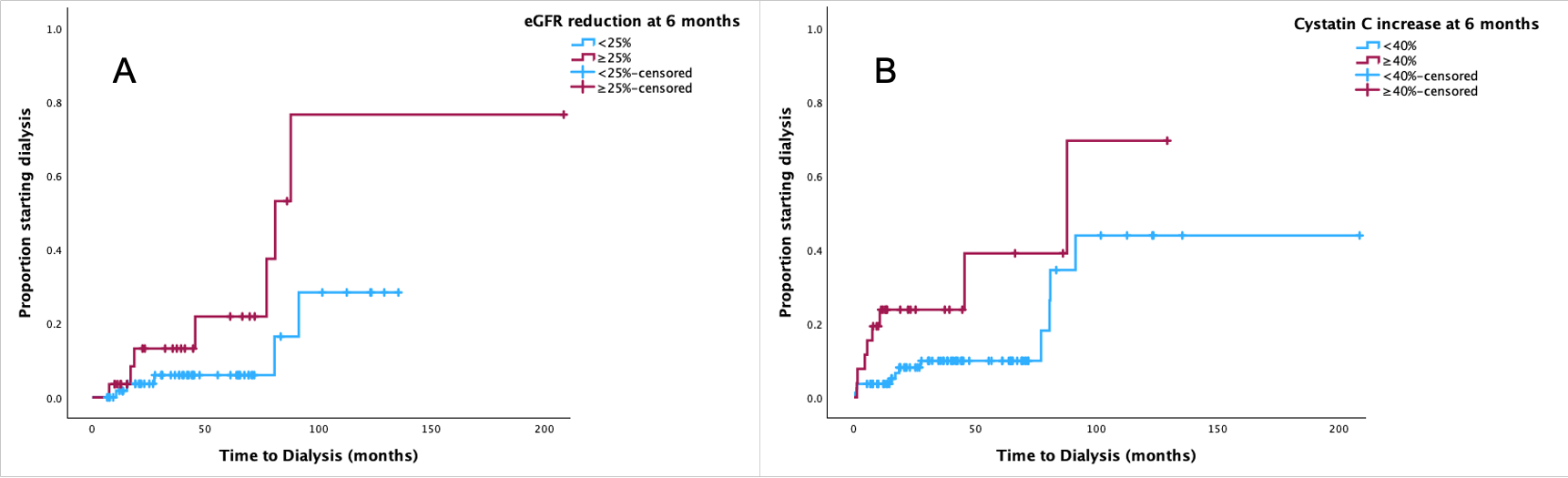


**Figure S5.** Time to dialysis for patients with renal PD (eGFR CKD-EPI decreased ≥25%) (A), and for patients with an increase in Cystatin C ≥40% (B).

**Table S6.** ROC analysis for prognostic performance of eGFR equations at 6-month landmark analysis.

|  | **ROC AUC** | **Std. Err.** | **95% CI** |
| --- | --- | --- | --- |
| eGFR CKD-EPI | 0.847 | 0.079 | 0.692-1 |
| eGFR MDRD | 0.847 | 0.082 | 0.687-1 |
| eGFRcys | 0.858 | 0.070 | 0.720-1 |
| eGFRcr-cys | 0.866 | 0.073 | 0.723-1 |
| eGFRcys-ns | 0.854 | 0.066 | 0.724-1 |


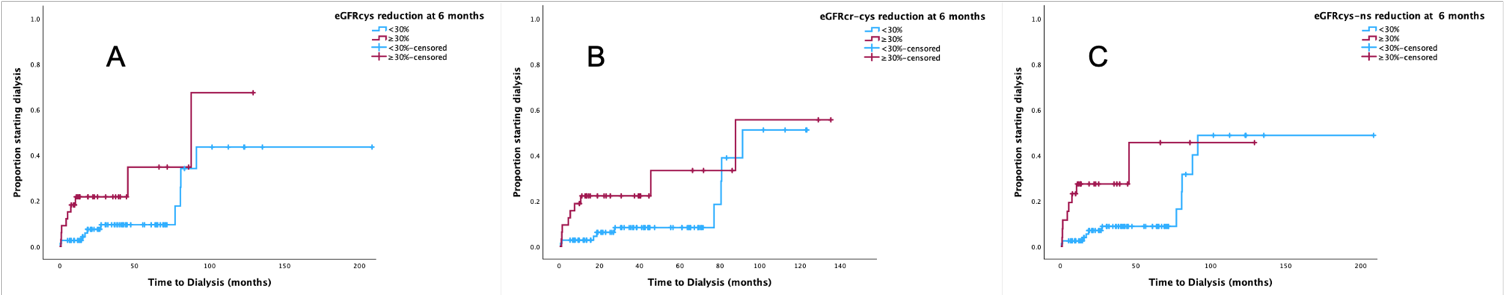


**Figure S6.** Time to dialysis for patients with a reduction in cystatin-based eGFR ≥30% vs those without reduction or with increase in eGFR.

**
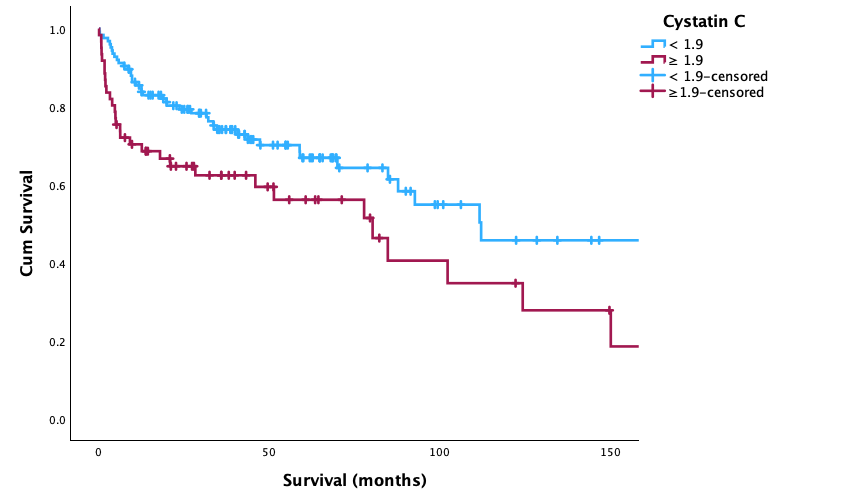
Figure S7.** OS according to baseline cystatin with cutoff > 1.9 mg/L.

**Results based on MDRD eGFR formula**


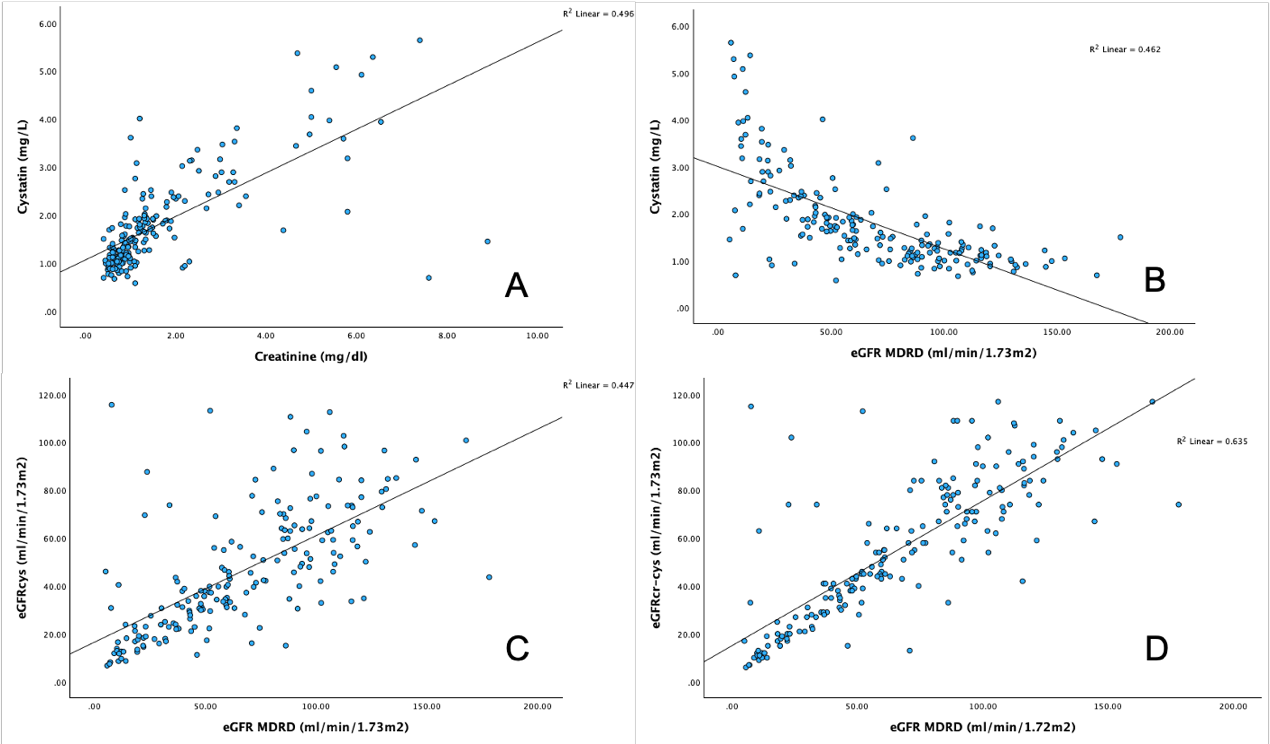


**Figure S8.** Correlation of baseline Cystatin C with creatinine (A) and eGFRcr MDRD (B). Correlation of eGFRcys (C) and eGFRcr-cys (D) with eGFRcr MDRD.

**Table S7.** Multivariate analysis including renal stage (using MDRD eGFR for creatinine and Cystatin C as continuous variable (Model 1) and as categorical variable (Model 2&3).

| **Multivariate analysis** |  |  |  |
| --- | --- | --- | --- |
| **Model 1** |  |  |  |
| Renal stage 1 | 1 (ref) |  |  |
| Renal stage 2 | 6.799725 | 0.9082154-50.90891 | 0.062 |
| Renal stage 3 | 12.85382 | 1.606775-102.8275 | **0.016** |
| Cystatin C | 2.044337 | 1.537121-2.718922 | **<0.0001** |
| **Model 2** |  |  |  |
| Renal stage 1 | 1 (ref) |  |  |
| Renal stage 2 | 7.352835 | 0.9825568-55.02397 | 0.052 |
| Renal stage 3 | 13.97761 | 1.743557-102.8275 | **0.013** |
| Cystatin C > 1.9mg/L | 4.002848 | 1.839208-8.711786 | **<0.0001** |
| **Model 3** |  |  |  |
| Proteinuria > 5000 mg/24h | 2.636428 | 1.161-5.987 | **0.021** |
| eGFR MDRD < 50 mL/min/1.73m^2^ | 1.794941 | 0.683-4.716 | 0.235 |
| Cystatin C > 1.9 mg/L | 4.437967 | 1.662-11.851 | **0.003** |

Table S8. Multivariate analysis for time to dialysis at 6-month landmark using eGFR MDRD formula.

| **Multivariate analysis** |  |  |  |
| --- | --- | --- | --- |
| **Model 1** |  |  |  |
| Serum albumin at 6 months, g/dl | 0.335 | 0.137-0.824 | **0.017** |
| eGFR **(MDRD)**at 6 months, ml/mL/1.73m^2^ | 0.990 | 0.956-1.043 | 0.950 |
| Cystatin C at 6 months, mg/L | 3.530 | 1.156-10.779 | **0.027** |
| **Model 2** |  |  |  |
| Renal PD at 6 months | 1.900 | 0.496-7.275 | 0.348 |
| Cystatin C at 6 months, mg/L | 3.108 | 1.614-5.984 | **<0.001** |
| **Model 3** |  |  |  |
| Renal response at 6 months | 1.090 | 0.297-4.001 | 0.897 |
| Cystatin C at 6 months, mg/L | 3.602 | 1.903-6.820 | **<0.001** |
| **Model 4** |  |  |  |
| RenalPD at 6 months | 2.255 | 0.576-8.831 | 0.243 |
| Cystatin C baseline | 2.770 | 1.293-5.937 | **0.009** |
| Cystatin C increase >1mg/L | 6.731 | 1.219-37.155 | **0.029** |

**References**

1. Levey AS, Bosch JP, Lewis JB, Greene T, Rogers N, Roth D. A more accurate method to estimate glomerular filtration rate from serum creatinine: a new prediction equation. Modification of Diet in Renal Disease Study Group. *Ann Intern Med.* 1999;130(6):461-470.

2. Inker LA, Eneanya ND, Coresh J, et al. New Creatinine- and Cystatin C–Based Equations to Estimate GFR without Race. *New England Journal of Medicine.* 2021;385(19):1737-1749.

3. Inker LA, Schmid CH, Tighiouart H, et al. Estimating Glomerular Filtration Rate from Serum Creatinine and Cystatin C. *New England Journal of Medicine.* 2012;367(1):20-29.

4. Pottel H, Björk J, Rule AD, et al. Cystatin C-Based Equation to Estimate GFR without the Inclusion of Race and Sex. *N Engl J Med.* 2023;388(4):333-343.
